# Wheat growth parameters prediction based on dual output Bayesian neural network using multi-modal information

**DOI:** 10.3389/fpls.2026.1871483

**Published:** 2026-06-26

**Authors:** Ke Xu, Dahui Cao, Qinyue Tai, Haiting Wang, Jun Ni, Yaocong Hu, Huicheng Yang

**Affiliations:** 1 School of Integrated Circuits, Anhui Polytechnic University, Wuhu, China; 2 Anhui Provincial Key Laboratory of Advanced Detection and Intelligent Sensing, Wuhu, China; 3 School of Mechanical and Automotive Engineering, Shanghai University of Engineering Science, Shanghai, China; 4 School of Electrical Engineering, Anhui Polytechnic University, Wuhu, China

**Keywords:** Bayesian neural network, dual output prediction, leaf area index (LAI), leaf nitrogen accumulation (LNA), multimodal information, wheat

## Abstract

**Introduction:**

eaf area index (LAI) and leaf nitrogen accumulation (LNA) are key indicators of wheat growth and nitrogen nutritional status. However, existing prediction methods predominantly rely on single-modal information and single-output models, limiting their ability to characterize the complex structural and physiological traits of crops. This study aimed to develop a multimodal learning framework for the simultaneous and accurate prediction of wheat LAI and LNA.

**Methods:**

Spectral, image, and canopy structural features were extracted from wheat canopies across different cultivars, nitrogen treatments, and growth stages. A canopy height correction-based preprocessing method was developed to improve the extraction of structural features. A Dual-Output Bayesian Neural Network (DO-BNN) was then constructed to simultaneously predict LAI and LNA. In addition, an Extreme Sample Mining (ESM) strategy and a joint loss function were introduced to strengthen the learning of complementary information across modalities and the intrinsic correlation between the two target variables.

**Results:**

The DO-BNN achieved its best predictive performance when all feature modalities were fused. The coefficients of determination (R²) for LAI and LNA were 0.89 and 0.77, respectively, while the corresponding relative root mean square errors (RRMSEs) were 0.15 and 0.35. Compared with single-modal and conventional single-output approaches, the proposed method provided more accurate and robust predictions of both wheat growth parameters.

**Discussion:**

The results demonstrate that integrating spectral, image, and structural information can improve the characterization of wheat canopy traits. By jointly modeling LAI and LNA, the DO-BNN effectively exploited the physiological relationship between crop growth and nitrogen accumulation. The proposed framework provides a promising approach for the high-accuracy, collaborative monitoring of wheat growth and nitrogen nutritional status.

## 1 Introduction

Wheat is one of the most important food crops worldwide, with an annual production of approximately 700 million tons, providing about 20% of the calories and protein consumed by humans globally, and playing a vital role in food security, economic development, and human nutrition (FAO, 2024). The real-time, rapid, accurate, and non-destructive acquisition of key wheat growth parameters is of great significance for optimizing field management and improving yield and quality. In recent years, with the development of remote sensing technology, the extraction of canopy spectral, textural, and structural features from imaging and non-imaging information, followed by the construction of crop growth parameter prediction models, has become an important technical approach for wheat growth monitoring and assessment (Wang et al., 2025). Among these parameters, leaf area index (LAI) and leaf nitrogen accumulation (LNA) reflect crop canopy structural status and nitrogen nutritional level, respectively, and are key indicators for evaluating crop growth condition and yield potential (Tao et al., 2026). With the continuous improvement in multi-source data acquisition and machine learning methods, the inversion of crop physiological parameters based on remote sensing information has become an important research direction in precision agriculture (Song et al., 2025).

Among various remote sensing features, vegetation indices constructed from visible and near-infrared bands have been widely used for crop growth parameter estimation because of their high sensitivity to chlorophyll content, canopy structure, and biomass. Previous studies have developed a variety of classical vegetation indices based on sensitive bands, including the Normalized Difference Vegetation Index, Ratio Vegetation Index, and Difference Vegetation Index, and have achieved good performance in the estimation of wheat LAI and LNA (Yan et al., 2025). Based on these spectral features, related studies have achieved effective prediction of LAI and LNA, for example, by constructing novel vegetation indices to improve the stability and accuracy of wheat LAI estimation (Li et al., 2025), or by selecting characteristic bands to accurately monitor the nitrogen nutritional status of wheat (Chen et al., 2025). However, canopy spectral reflectance is influenced not only by plant biochemical components, but also by leaf arrangement, canopy geometric structure, and observation geometry, thus exhibiting pronounced anisotropic characteristics (Kong et al., 2025; Yang et al., 2026). Therefore, relying solely on single-modal spectral information makes it difficult to comprehensively characterize the complex physiological and structural states of crops, which in turn limits the stability and generalization ability of prediction models under complex conditions.

To overcome the limitations of using spectral information alone, multimodal approaches for crop growth parameter prediction have attracted increasing attention in recent years. By integrating image features with three-dimensional structural information, these methods can complement crop morphological characteristics while enhancing the comprehensive characterization of crop growth status (Gao et al., 2025; Shu et al., 2022). Existing studies have shown that multi-source data fusion can effectively improve the representation ability of models for complex agricultural systems. For example, the fusion of spectral and depth images can significantly improve the prediction accuracy of nitrogen accumulation in wheat, while the integration of spectral, structural, and thermal infrared features from high-resolution UAV imagery can also achieve high-accuracy estimation of LAI (Wu et al., 2022). Further studies have indicated that three-dimensional structural information, such as the canopy height model, helps characterize the spatial distribution of the canopy, thereby improving the estimation accuracy of crop structural parameters (Li et al., 2021). Nevertheless, most existing studies still construct models around a single target variable, that is, they independently predict either LAI or LNA. Although this modeling strategy can achieve good performance for specific tasks, it often overlooks the potential intrinsic relationships among different growth parameters, thereby limiting feature sharing and information utilization efficiency and leaving room for further improvement in model generalization under complex conditions.

In fact, different crop growth parameters often exhibit significant physiological coupling relationships, which originate from the coordinated mechanisms of structural formation and nutrient regulation during crop growth (Han et al., 2025). LAI determines the canopy’s ability to intercept light energy and is closely related to nitrogen uptake and allocation, while nitrogen status further regulates leaf growth and canopy structure by affecting chlorophyll content and photosynthetic efficiency (Han et al., 2023). Based on this intrinsic relationship, joint modeling is expected to characterize crop growth mechanisms more accurately through feature sharing and collaborative learning. In recent years, multi-task learning has gradually attracted attention in agricultural remote sensing and crop parameter inversion. Relevant studies have shown that jointly estimating multiple physiological or structural parameters can effectively exploit the coupling relationships among parameters, thereby improving model performance and stability (Bahrami et al., 2022). Previous studies have verified the effectiveness of multi-objective optimization in the joint inversion of chlorophyll content and LAI, indicating that joint modeling can simultaneously account for the interactions among parameters and improve the accuracy of multi-parameter inversion (Du et al., 2023; Yue et al., 2024). However, existing joint modeling studies have mainly focused on single-modal or limited-modal information. For parameters such as LAI and LNA, which simultaneously reflect structural and nutritional attributes, systematic research is still lacking on how to simultaneously exploit modal complementarity and parameter coupling under multimodal conditions. In addition, multimodal feature fusion may introduce information redundancy, information conflicts, and scale inconsistency, thereby increasing the difficulty of model learning and affecting the stability and reliability of prediction results.

Based on the above research background, this study proposes a Dual Output Bayesian Neural Network (DO-BNN) model based on multimodal information for the simultaneous prediction of wheat LAI and LNA. Using multimodal features extracted from spectral, two-dimensional image, and depth image data as inputs, the proposed method achieves joint modeling of LAI and LNA by constructing a DO-BNN. In addition, Bayesian inference is incorporated to quantify model uncertainty, thereby improving prediction accuracy while enhancing model robustness. Specifically, this study first extracts canopy spectral and image features of wheat under different cultivars, nitrogen treatments, and growth stages, and proposes a height feature extraction method based on Canopy Height Correction Method (CHCM) to strengthen the representation of canopy structural information. Next, a multimodal DO-BNN is constructed to achieve the simultaneous prediction of LAI and LNA. Finally, an Extreme Sample Mining (ESM) strategy and a joint loss function are introduced to enable the model to more fully learn the complementarity and consistency among different modalities, while also strengthening the intrinsic relationships between the two outputs.

The methodological novelty of this study can be summarized from three aspects. Compared with conventional multi-output regression and multi-task learning methods, the proposed DO-BNN has three main methodological contributions. First, it integrates spectral, RGB texture, and depth-derived structural features to jointly characterize the physiological and structural status of wheat canopies. Second, Bayesian inference is embedded into the dual-output neural network to simultaneously estimate the predicted values and predictive uncertainty of LAI and LNA. Third, an ESM strategy is designed within the dual-output framework to strengthen the learning of difficult and extreme samples while considering the coupling relationship between LAI and LNA. Therefore, the proposed method is not a simple combination of multimodal feature fusion and multi-output regression, but a probabilistic collaborative prediction framework that jointly exploits modal complementarity, output correlation, and sample-level uncertainty.

## 2 Materials and methods

### 2.1 Experiment design

**Figure 1:**
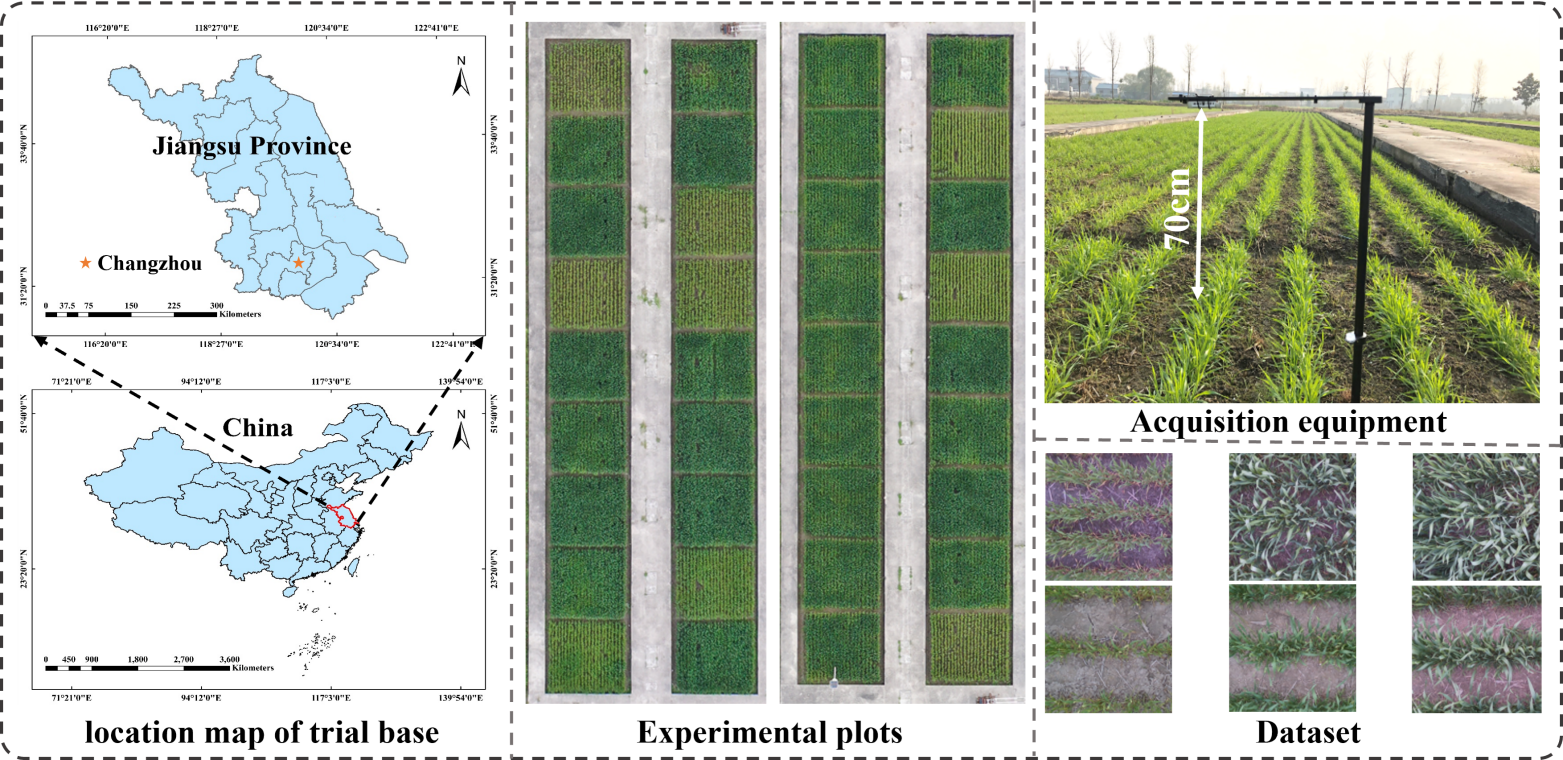
Experiment site and data acquisition.

From 2022 to 2024, continuous wheat field experiments were conducted in Changzhou, Jiangsu Province, China (31°47′N, 119°57′E) to investigate the effects of different management practices and cultivar types on the monitoring of wheat growth parameters. The experimental design is shown in **Figure 1**. In Experiment 1 (2022), the spreading-type cultivar Sumai 8 (V1) was used, with three nitrogen fertilizer levels (0, 180, and 360 kg/hm^2^) and three row spacing treatments (50 cm, 35 cm, and 20 cm). In Experiment 2 (2023), the erect-type cultivar Shengxuan 6 (V2) was used, and the nitrogen fertilizer and row spacing treatments were the same as those in the previous year. In Experiment 3 (2024), two cultivar types were used simultaneously, namely Yangmai 16 (V1, spreading type) and Yangmai 23 (V2, erect type). Under a uniform row spacing of 20 cm, inter-cultivar comparisons were conducted, while the nitrogen fertilizer treatments were kept the same as those in the previous two years. In all experiments, the nitrogen fertilizer application ratio was basal fertilizer: jointing fertilizer = 5: 5, with 135 kg/hm^2^ of P_2_O^5^ and 220 kg/hm^2^ of K_2_O applied together with the basal fertilizer. The plot area was uniformly 30 m^2^ (6 m × 5 m). Multimodal data acquisition and growth parameter measurements were carried out at the jointing stage (S1), booting stage (S2), and heading stage (S3) of wheat, respectively.

### 2.2 Data acquisition

#### 2.2.1 Multimodal information acquisition

Wheat canopy spectral data were acquired using a portable hyperspectral spectrometer, ASD FieldSpec HandHeld 2 (Analytical Spectral Devices, Boulder, CO, USA). Measurements were conducted at midday on sunny days (10:30 h–14:00 h), with the spectrometer positioned 1 m above the wheat canopy. For each plot and sampling date, spectral measurements, RGB-D image acquisition, LAI measurement, and destructive sampling for LNA determination were conducted on the same day. Three representative sample points were randomly selected in each plot for non-destructive measurements. Spectral, RGB, depth, and LAI measurements were collected from the same representative plot area, and the average of the three sample points was taken as the plot-level spectral data. Canopy RGB-D images were synchronously collected vertically above the wheat canopy using an Intel^®^ RealSense™ Depth Camera D435i. The camera uses a built-in structured-light infrared projector to project light patterns onto the target. The infrared camera then captures phase information, which is converted into depth information by the processing unit. The D435i has an operating range of 0.3–10 m, a field of view of 69.4° × 42.5° × 77°, and both the depth and RGB images have a resolution of 1280 × 720 pixels. The central 720 × 720 pixel region was cropped for canopy structural feature extraction, and the average of the three sample points was used as the canopy structural feature for each plot.

#### 2.2.2 Growth information acquisition

A continuous 50 cm section of wheat plants was selected from each plot for destructive sampling. After separating the aboveground organs of wheat plants, the samples were placed into paper bags, deactivated at 105 °C for 30 min, and then oven-dried at 80 °C to constant weight. The leaf dry weight (LDW, g m^−2^) was obtained by weighing the dried wheat leaves. The dried wheat leaves were then ground into powder, and the leaf nitrogen concentration (LNC, g kg^−1^) of the target wheat plants was determined using the Kjeldahl method. Leaf nitrogen accumulation (LNA, g m^−2^) was calculated from LDW and LNC as follows in Equation 1:

(1) LNA=LDW×LNC,

The LAI of the target plots was measured using an LAI-2200C canopy analyzer (Li-Cor, Lincoln, NE, USA). Three sample points were randomly selected in each plot for measurement, and the average value was taken as the LAI of that plot.

### 2.3 Multi-modal feature extraction

#### 2.3.1 Spectral feature extraction

Although hyperspectral data contain rich spectral information, directly using full-spectrum features with a limited sample size may increase feature redundancy and overfitting risk. Therefore, this study focused on representative sensitive bands related to red-edge and near-infrared responses. The bands at 730 nm and 815 nm were selected because previous studies have shown that they are sensitive to canopy chlorophyll, leaf area, and nitrogen nutritional status (Li et al., 2020; Feng et al., 2008). These two bands were then used to construct NDRE and RVI.

(2) $$NDRE=\frac{\left(R_{815}-R_{730}\right)}{\left(R_{815}+R_{730}\right)},$$

(3) $$RVI=\frac{R_{730}}{R_{815}},$$

where *R*_730_ and *R*_815_ represent the reflectance of wheat at 730 nm and 815 nm in Equations 2, 3, respectively.

#### 2.3.2 Image feature extraction

Texture information reflecting canopy spatial distribution characteristics can be extracted from wheat canopy RGB images. To make better use of RGB color information, this study adopted the Color Co-occurrence Matrix (CCM). CCM extends the gray-level co-occurrence matrix by quantifying spatial correlations among different color channels. It can therefore describe both color and texture properties of canopy images. Specifically, the acquired canopy images were first converted from the RGB color space to the HSV color space, and on this basis, four classical Haralick texture features were extracted: Entropy (ENT), Angular Second Moment (ASM), Contrast (CON), and Correlation (COR) (Maheswari et al., 2011). These four texture features characterize the structural and distributional properties of crop canopy images from different perspectives. Among them, ENT reflects the randomness of the image gray-level distribution, with larger values indicating more complex and irregular textures. ASM is used to measure the uniformity and energy of an image, and a larger value indicates a smoother texture and a more stable structure. CON describes the magnitude of gray-level variation and can characterize canopy brightness differences and boundary clarity. COR reflects the spatial correlation among image gray values and is used to characterize texture directionality and structural consistency.

#### 2.3.3 Structural feature extraction

A depth camera measures the distance from the camera to the target surface along the line of sight. This distance is not equivalent to the true vertical height relative to the ground. Therefore, directly using depth values to estimate canopy height may cause large errors when the ground is uneven or the camera is tilted. To improve the accuracy of canopy structural feature extraction, this study proposes a Canopy Height Correction Method (CHCM). First, the canopy image was reconstructed into a three-dimensional point cloud by combining the depth image with the camera intrinsic parameters. Then, a ground reference plane was fitted using the least-squares method, and the orthogonal distance from each canopy point to the fitted plane was calculated to effectively eliminate the effects of camera view angle variation and ground inclination, thereby more accurately reflecting the actual spatial height distribution of the wheat canopy. On this basis, the Canopy Volume Index (CVI) and canopy height features were further extracted from the preprocessed height image. Specifically, the CVI was calculated by spatially integrating the canopy height at each pixel with the ground as the reference and was used to characterize the overall spatial occupancy of the canopy. The canopy height features were quantitatively described using the coefficient of variation of height and height percentile statistics. All structural features are listed in **Table 1**.

**Table 1:** Canopy structural features obtained based on canopy height images and relevant description.

Structural feature	Description
H_mean	Mean value of canopy height
H_CV	Coefficient of variation of canopy height
HP95th	95th percentile canopy height
CVI	Canopy volume index

### 2.4 BNN model combining dual output and ESM

#### 2.4.1 Designed dual output BNN architecture

A neural network can be regarded as a conditional distribution model *P*(*y* | *x*,*w*), where the input (*x*) yields the predictive distribution of (*y*), and (*w*) denotes the weights in the neural network. In regression problems, *P*(*y* | *x*,*w*) is a Gaussian distribution with a fixed standard deviation, and its mean is taken as the prediction result. In conventional neural networks, the weights (*w*) are fixed values and are learned through Maximum Likelihood Estimation (MLE). However, models trained by MLE are usually prone to overfitting, especially when the size of the training data is smaller than the number of trainable parameters. To address this problem, Bayesian estimation introduces prior assumptions. Unlike the point estimation of the weights (*w*) in conventional neural networks, all weights in a BNN are represented by probability distributions, thereby introducing uncertainty into the predictions of the neural network. Specifically, by Bayesian estimation, the posterior distribution *P*(*w* | *D*) of (*w*) is obtained. At this point, the probabilistic model for predicting ( $\hat{y}$) from input ( $\hat{x}$) based on *w* can be written as Equation 4:

(4) $$P(\hat{y}|\hat{x})=E_{P(w|D)}[P(\hat{y}|\hat{x},w)],$$

where *P*(*w* | *D*) denotes the dataset, and each possible *w* in *P*(*w* | *D*) can be used to make a corresponding prediction. The mathematical expectation is taken as the average prediction value.

**Figure 2:**
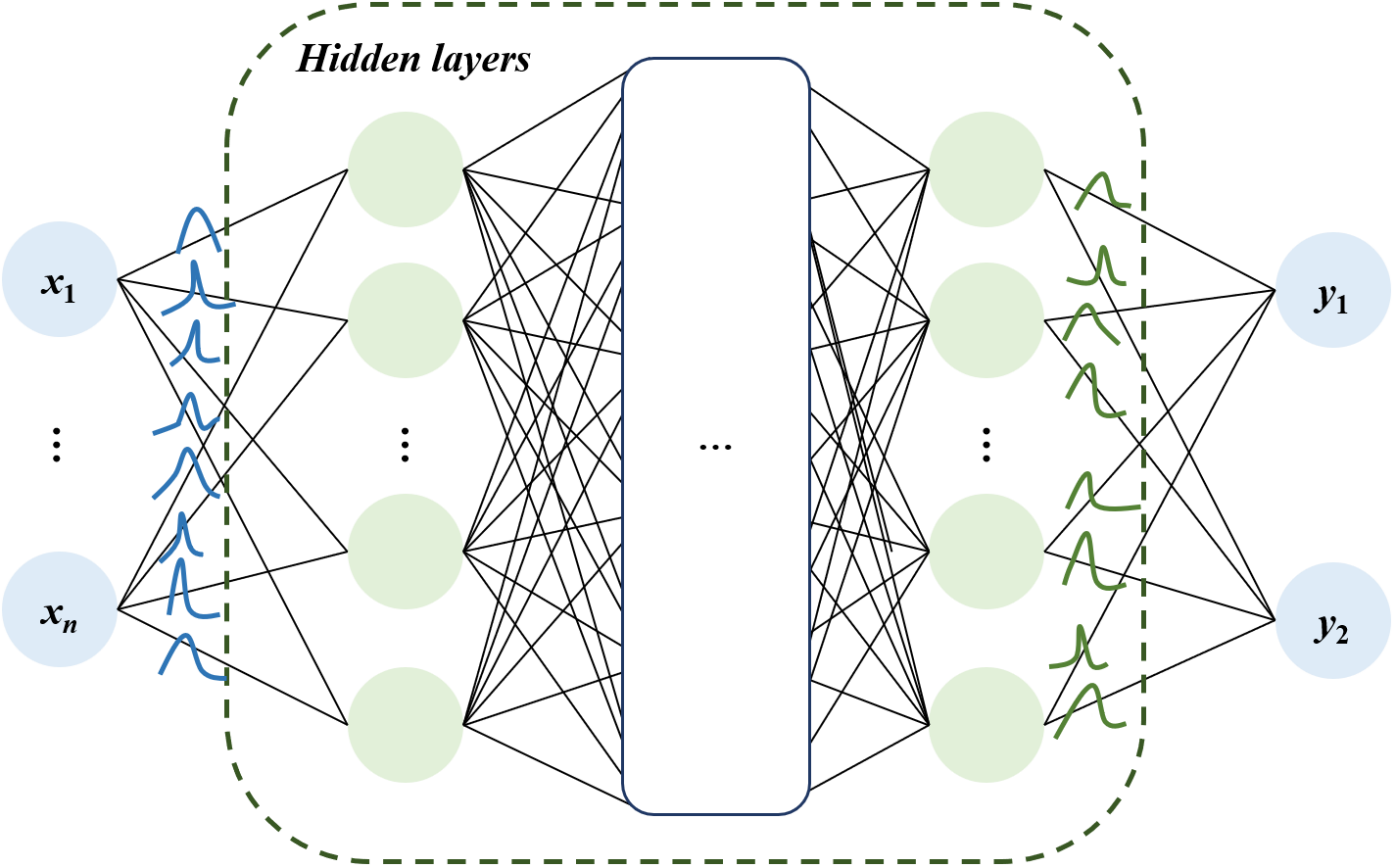
Structure diagram of dual output Bayesian neural network model.

Based on this, a DO-BNN model with ***L*** hidden layers was proposed in this study, as shown in the **Figure 2**. Assuming that the output responses of the BNN are both subject to Gaussian noise $\epsilon ∼N(0,\theta_{\sigma^{2}})$, and $\theta_{\sigma^{2}}$ denotes the variance parameter of the Gaussian noise, the output responses can be expressed as Equation 5:

(5) $${\hat{g}}_{t}(x)=f(x,\tau ;w)+\epsilon ,$$

where f(x,τ;w) denotes the τ-th(τ=1,2,⋯,T) output response in the BNN. Accordingly, the probabilistic model of the BNN can be expressed as Equation 6:

(6) $$p({\hat{g}}_{t}(x)|x,\theta )=N(f(x,\tau ;w),\theta_{\sigma^{2}}),$$

according to Bayes’ theorem, the joint probability distribution of the BNN model parameters $\theta =(w,\theta_{\sigma^{2}})$ and the observed dataset ***D*** can be expressed as the product of the prior distribution of the BNN model parameters and their likelihood over the available training samples, that is shown in Equation 7:

(7) $$\begin{array}{l} p(D,\theta )=p(\theta )p(D|\theta )\\ =p(w)p(\theta_{\sigma^{2}})\prod_{\tau =1}^{T}\prod_{\tau =1}^{D_{\tau }}N(y_{\tau }^{i}|f(x^{i},\tau ;w),\theta_{\sigma^{2}}), \end{array}$$

where *p*(*w*) represents the prior distribution of the mean parameters of the BNN, $p(\theta_{\sigma^{2}})$ represents the prior distribution of the variance of the BNN predictive outputs, *D_τ_* denotes the number of training samples corresponding to the *τ*-*th* output, and $y_{\tau }^{i}$ denotes the *τ*-*th* output of the *i*-*th* sample. After obtaining the posterior distribution $\theta^{i}\sim p(\theta |D)$ of the BNN parameters for the *i*-*th* sample, random sampling can be performed on the BNN model parameters, thereby yielding the probability distribution of multiple predictive outputs:

(8) $$P({\hat{g}}_{\tau }(x)|x,D)=\int_{\theta }p({\hat{g}}_{\tau }(x)|x,\theta )p(\theta |D)d\theta \approx \frac{1}{M}\sum_{i=1}^{M}p({\hat{g}}_{\tau }(x)|x,\theta^{i}),$$

where ***M*** is the number of randomly sampled samples. According to Equation 8, for any given input x*, the BNN can obtain the corresponding predictive mean and variance as follows:

(9) $$\mu ({\hat{g}}_{\tau }(x^{*})|D)=\frac{1}{M}\sum_{i=1}^{M}f(x^{*},\tau ;w^{i}),$$

(10) $$\sigma^{2}({\hat{g}}_{\tau }(x^{*})|D)=\frac{1}{M}\sum_{i=1}^{M}\left(f(x^{*},\tau ;w^{i}\right)-\mu ({\hat{g}}_{\tau }(x^{*})|D){)}^{2}+\theta_{\sigma 2}^{i},$$

According to Equations 9, 10, the BNN can simultaneously estimate the mean and variance of multiple outputs. In multi-output regression tasks, different outputs usually have different levels of prediction difficulty and uncertainty. Therefore, the BNN is well suited for solving multi-output regression problems.

Model implementation details were as follows. All input features were standardized using z-score normalization based on the mean and standard deviation of the training set. LAI and LNA were also standardized before model training and then inverse-transformed for performance evaluation. The DO-BNN consisted of an input layer, two Bayesian fully connected hidden layers with 32 and 16 neurons, respectively, and two output nodes corresponding to LAI and LNA. The ReLU function was used as the activation function. For each Bayesian layer, the weight prior was set as a zero-mean Gaussian distribution, $p(w)=N(0,\sigma_{p}^{2}),$ where σ*_p_* = 1. A mean-field Gaussian variational posterior *q*(*w*) was used to approximate the true posterior distribution, and the model was optimized by minimizing the negative evidence lower bound together with the joint regression loss. During training, 20 Monte Carlo samples were used to estimate the predictive distribution, and 100 stochastic forward passes were used during inference to calculate the predictive mean and uncertainty. The Adam optimizer was used with a learning rate of 0.001, batch size of 16, and a maximum of 800 epochs. Early stopping was applied based on the validation loss with a patience of 80 epochs.

#### 2.4.2 ESM based on active learning for dual output model

Sample Mining is a commonly used approach in machine learning. Its main idea is to mine samples with high loss during training (i.e., hard examples) and add them back into the dataset for retraining, so as to improve the predictive ability of the model. Based on this idea, this study proposes an ESM method based on active learning, which improves the construction of the initial model and the efficiency of subsequent optimization by automatically selecting key samples that contribute most to improving model accuracy. At the same time, the intrinsic relationships among different outputs in the dual-output model are fully considered during the process of ESM. The active-learning-based sample selection method selects at most three points in each iteration. By maximizing $\bar{\sigma }(x)$, the point with the largest average predictive variance can be selected, that is, the point that contributes the most to reducing the predictive variance of the multi-output model. $\bar{\sigma }(x)$ is the mean of the predictive variances corresponding to the *T* outputs, and is calculated as follows:

(11) $$\bar{\sigma }(x)=\frac{1}{M}\sum_{\tau =1}^{T}\sigma ({\hat{g}}_{\tau }(x)|D),$$

Because the nonlinearity of the functional relationship is usually higher at extreme points, identifying such points is important for improving model prediction accuracy. The optimal sample points are selected from the candidate extreme samples using *MAX*(*x*) and *MIN*(*x*), where *MAX*(*x*) denotes the samples that are likely to be local maxima among the ***T*** outputs, and *MIN*(*x*) denotes the samples that are likely to be local minima among the ***T*** outputs. Their calculation is as follows:

(12) $$MAX(x)=\underset{\tau (\tau =1,2,\cdots ,T)}{\max}\left\{\frac{\mu ({\hat{g}}_{\tau }(x)|D)+\beta_{\tau }^{\max}\sigma ({\hat{g}}_{\tau }(x)|D)}{\max(\mu ({\hat{g}}_{\tau }(x)|D))-\min(\mu ({\hat{g}}_{\tau }(x)|D))}\right\},$$

(13) $$MIN(x)=\underset{\tau (\tau =1,2,\cdots ,T)}{\min}\left\{\frac{\mu ({\hat{g}}_{\tau }(x)|D)+\beta_{\tau }^{\min}\sigma ({\hat{g}}_{\tau }(x)|D)}{\max(\mu ({\hat{g}}_{\tau }(x)|D))-\min(\mu ({\hat{g}}_{\tau }(x)|D))}\right\},$$

where $\beta_{\tau }^{\max}$ and $\beta_{\tau }^{\min}$ denote the adjustable parameters used in searching for the extreme values corresponding to the *τ*-*th* output, respectively. To eliminate the influence of scale effects among different outputs during the calculation process, normalization terms were added to the denominators of Equation 12, 13. To determine whether the constructed model had reached the convergence condition, the generalization error of the model on the validation dataset was used as the criterion. When the generalization error was smaller than the given threshold ζ, the model was considered to have converged.

The ESM procedure was conducted only on the training set to avoid information leakage from the independent test set. First, the initial DO-BNN was trained using the original training samples. Then, the predictive mean and variance of each training sample were estimated through Monte Carlo sampling. Candidate extreme samples were identified according to three criteria: high predictive uncertainty, potential local maximum response, and potential local minimum response, which were calculated using Equations 11–13. In each mining iteration, at most three samples were selected from the candidate pool. These selected samples were not added to the validation or test set; instead, they were emphasized during subsequent training by assigning them a larger sample weight of α = 2.0. The mining process was repeated for five iterations or until the validation error no longer decreased.

#### 2.4.3 Joint loss function for dual output model

For a dual-output regression problem, the loss function needs to take into account both regression tasks and the uncertainty modeling in the Bayesian network. Assuming that the predictions of the dual-output model are ${\hat{y}}_{1}$ and ${\hat{y}}_{2}$, and the corresponding ground-truth values are *y*_1_ and *y*_2_, respectively, the root mean square error function is adopted as the regression loss, that is shown in Equation 14:

(14) $$MSE=\frac{1}{N}\sum_{i}^{N}{\left[({\hat{y}}_{i}-y_{i})\right]}^{2},$$

Then, the loss functions for different regression tasks can be formulated as Equation 15:

(15) $$L_{reg}=\lambda_{1}\cdot MSE_{1}+\lambda_{2}\cdot MSE_{2},$$

where λ_1_ and λ_2_ denote the weights of the loss terms for different regression tasks, respectively. The Bayesian neural network estimates model uncertainty by sampling from the posterior distribution; therefore, uncertainty modeling needs to be incorporated into the loss function. Since it is difficult to solve the posterior distribution *P*(*w* | *D*) directly using Bayes’ theorem in high-dimensional tasks, variational inference is typically employed to introduce an approximate distribution *q*(w) to replace the true posterior distribution. In the design of the loss function, the Kullback–Leibler divergence is used to measure the difference between the approximate distribution and the prior distribution *p*(w). At the same time, it can serve as a regularization term to constrain the weight distribution of the model and avoid overfitting. Therefore, the loss function of the dual-output BNN can be summarized as Equation 16:

(16) $$L_{total}=\lambda_{1}\cdot MSE_{1}+\lambda_{2}\cdot MSE_{2}+\lambda_{3}\cdot KL(q(w)||p(w))$$

where λ_3_ is a hyperparameter used to represent the weight of the regularization term. In the joint loss function, λ_1_, λ_2_, and λ_3_ were set to 1.0, 1.0, and 1 × 10^−4^, respectively. Since LAI and LNA were standardized before training, equal weights were assigned to the two regression losses. The KL divergence weight was set to 1 × 10^−4^ to provide moderate Bayesian regularization while avoiding excessive constraint on model fitting. The value of λ_3_ was selected based on preliminary experiments using the validation set.

### 2.5 Evaluation metrics

A total of 216 samples were obtained from all experiments. Among them, the experimental data from the first and second years were used as the training and validation set (training: validation = 8:2), comprising 162 samples, while the experimental data from the third year were used as the test set, comprising 54 samples. The models were evaluated using (R2) (Coefficient of Determination) and RRMSE (Relative Root Mean Square Error), which were calculated as Equations 17, 18:

(17) $$R^{2}=1-\frac{\sum_{i=1}^{N}{\left(y_{i}-{\hat{y}}_{i}\right)}^{2}}{\sum_{i=1}^{N}{\left(y_{i}-\bar{y}\right)}^{2}},$$

(18) $$RRMSE=\frac{\sqrt{\frac{1}{N}\sum_{i=1}^{N}{\left(y_{i}-{\hat{y}}_{i}\right)}^{2}}}{\bar{y}},$$

where *y_i_*, ${\hat{y}}_{i}$, and $\bar{y}$ represent the predicted values, measured values, and mean measured values of wheat LNA and LAI, respectively, and ***N*** denotes the number of samples.

## 3 Results

### 3.1 Relationship between LAI–LNA correlation and VI-based prediction performance

**Figure 3:**
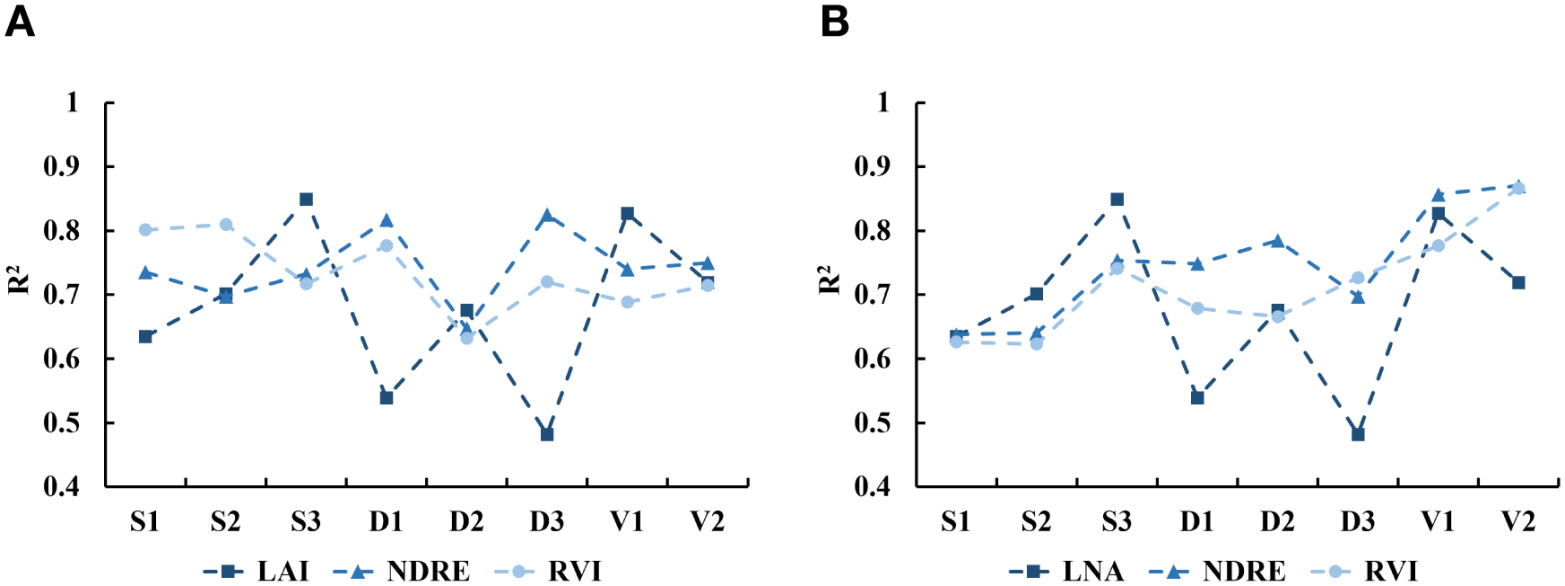
Effect of LAI–LNA correlation on VI-based prediction performance. **(A)** LAI prediction performance **(B)** LNA prediction performance.

**Figure 3** shows the prediction results of LAI and LNA based on spectral features under different cultivars, multiple growth stages, and density treatments. **Figures 3A, B** take the fitting results of LAI and LNA as the baseline, respectively, and compare the predictive performance of NDRE and RVI under different treatment conditions. Overall, changes in the correlation between LAI and LNA affected the prediction performance based on spectral indices to some extent, but this effect was not consistent for LAI and LNA. For LAI prediction, the predictive accuracies of NDRE and RVI were relatively stable. When the correlation coefficient increased from 0.48 to 0.84, the predictive accuracies of NDRE and RVI increased from 0.69 and 0.72 to 0.75 and 0.74, respectively, with only small overall differences, indicating that spectral features had a relatively stable characterization ability for LAI. In contrast, the prediction results for LNA showed a more complex response to changes in the LAI–LNA correlation. When the correlation coefficient was 0.84, the predictive accuracies of NDRE and RVI were 0.73 and 0.71, respectively; when the correlation coefficient decreased to 0.48, they increased to 0.82 and 0.72, respectively, with NDRE showing a more pronounced change. This indicates that, for LNA, the predictive ability of spectral features is more easily affected by the combined changes in canopy structure and nitrogen status under different treatment conditions. These results suggest that changes in the correlation between LAI and LNA influence the predictive performance based on spectral indices, and that the relationship between LNA and spectral features is more susceptible to interference from changes in crop structural status, making it difficult to achieve stable characterization using spectral indices alone.

### 3.2 Feature selection and analysis

**Figure 4:**
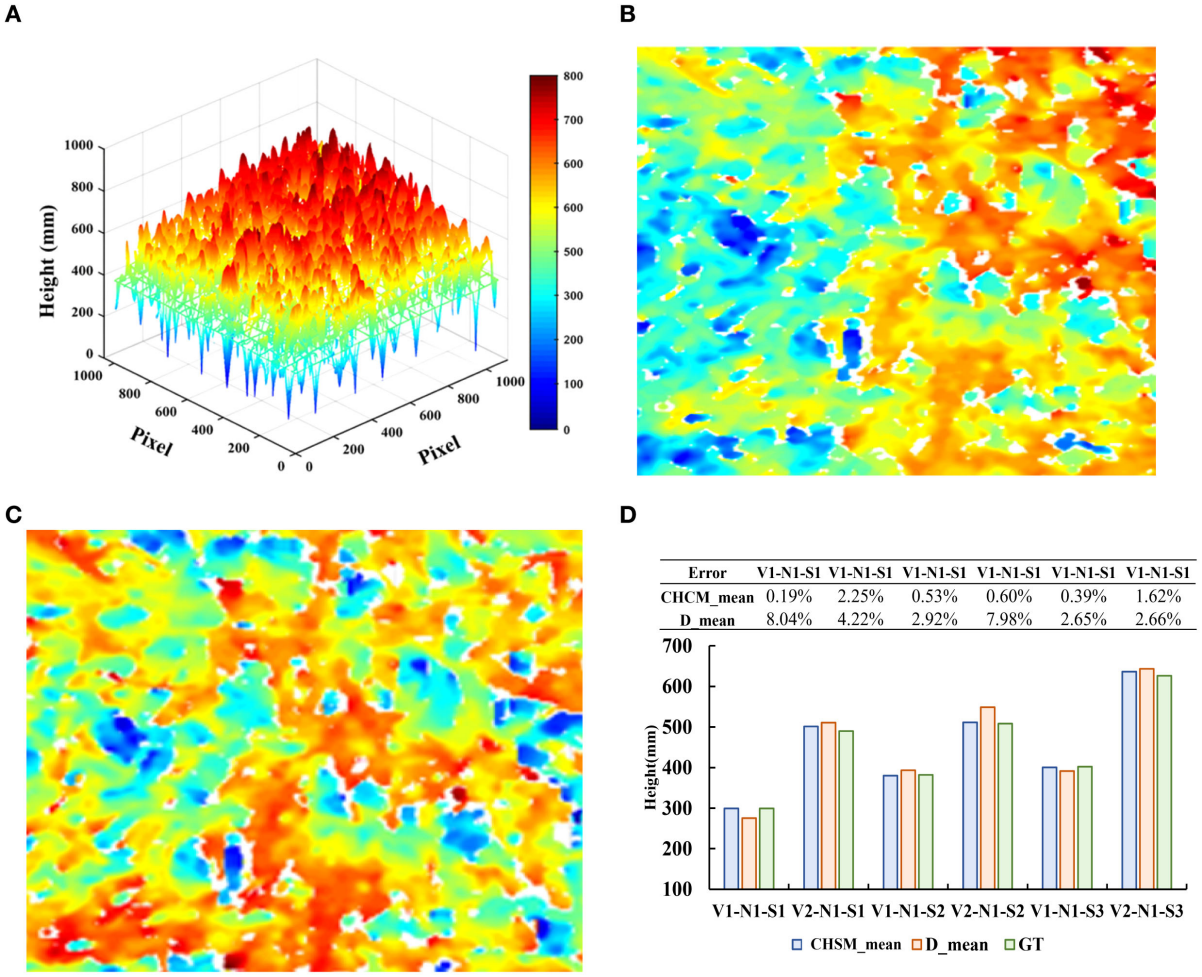
Results and validation of canopy height correction using CHCM. **(A)** 3D point cloud reconstruction and ground plane fitting. **(B)** Original depth image. **(C)** CHCM-corrected canopy height map. **(D)** Comparison of canopy mean height extracted by different methods and ground truth.

**Figure 4** presents the results of canopy height correction based on CHCM and its validation process. Specifically, **Figure 4A** shows the reconstruction of the depth image into a three-dimensional point cloud and the fitting of the ground reference plane; **Figures 4B, C** the original depth image and the CHCM-corrected result, respectively; and **Figure 4D** compares the differences between canopy mean height extracted by different methods and the measured Ground Truth (GT). Here, CHCM_mean and D_mean denote the canopy mean height extracted based on CHCM and the height mean directly calculated from the original depth image, respectively. For clarity, representative samples of two wheat cultivars at different growth stages under the same nitrogen level were selected. The results showed that, at all growth stages, the erect-type cultivar (V2) consistently exhibited greater canopy height than the spreading-type cultivar (V1). Meanwhile, for the same cultivar, canopy height gradually increased as growth progressed. To quantitatively evaluate the accuracy of different height-mean calculation methods, the ratio of the error to the true value was used for statistical analysis. The results showed that the relative errors of CHCM_mean were all below 2.5%, with the minimum error being only 0.19%. In contrast, the relative errors of D_mean were all higher than 2.5%, with the maximum error reaching 8.04%. These results indicate that the height values extracted based on CHCM can more accurately reflect the true canopy height characteristics of wheat. Therefore, all subsequent structural features were extracted from depth images preprocessed by CHCM.

**Figure 5:**
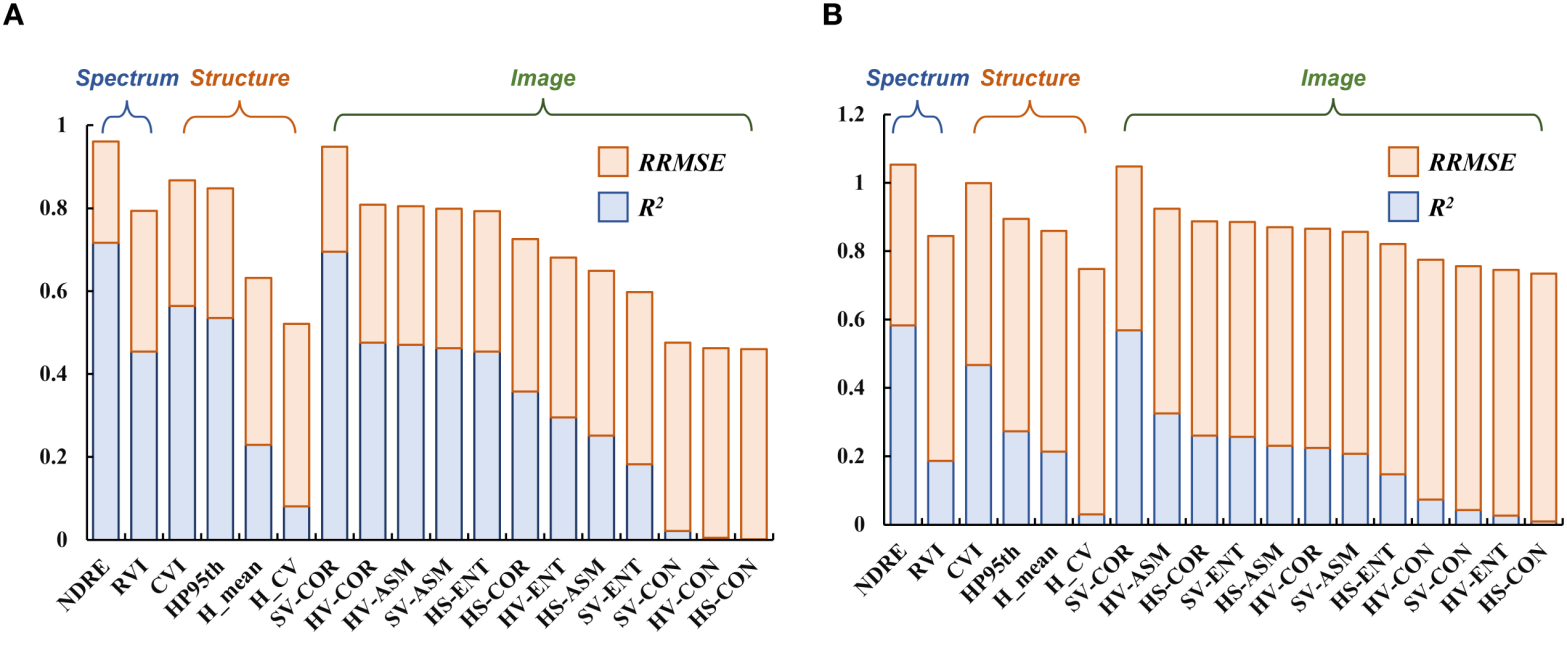
Modeling results of LAI and LNA based on multimodal features. **(A)** LAI modeling results based on the two-year dataset. **(B)** LNA modeling results based on the two-year dataset.

The representative features of different modalities were selected using a two-step strategy. First, candidate spectral, structural, and image texture features were constructed based on prior agronomic knowledge and previous studies. **Figures 5A, B** show the modeling results of LAI and LNA based on spectral features, canopy structural features, and image texture features. Overall, all types of features achieved better predictive performance for LAI than for LNA. Among the spectral features, NDRE performed best, with (*R^2^*) values of 0.72 and 0.58 and RRMSE values of 0.24 and 0.47 for LAI and LNA, respectively. Among the canopy structural features, the CVI extracted from depth images showed the best performance, with (*R^2^*) values of 0.53 and 0.27 and RRMSE values of 0.31 and 0.62 for LAI and LNA, respectively. Among the image texture features, SV_COR achieved the best results, with (*R^2^*) values of 0.70 and 0.57 and RRMSE values of 0.25 and 0.48 for LAI and LNA, respectively. Second, the predictive performance of each candidate feature was evaluated using the two-year training dataset. Since a large number of candidate features were extracted from different modalities, NDRE, CVI, and SV_COR were selected as representative features of the spectral, structural, and texture modalities, respectively, for further in-depth analysis of the roles of different modal features in LAI and LNA prediction tasks.

### 3.3 Analysis of prediction results of single-output models based on different modalities

To analyze the effects of different modal features on the prediction performance of LAI and LNA, comparative experiments were conducted using commonly used traditional single-output machine learning models, and the hyperparameter settings of these models are listed in **Table 2**. **Tables 3**, **4** present the prediction results of single-output models based on LAI and LNA data under different combinations of modal features, respectively. Here, the Prediction pipeline indicates that the LAI or LNA experimental data from the first two years were used as the training set, while the LAI and LNA data from the third year were used as the test set.

**Table 2:** Hyperparameter settings of different machine learning models.

Model	Hyperparameters
Stepwise	Max step=1000
BP	Two hidden layers, ReLU activation, Epoch=1000
SVR	Gaussian kernel function
Tree	Medium tree, Minimum samples leaf=12
BNN	Two hidden layers, ReLU activation, Epoch = 800, Gaussian prior, variational inference

**Table 3:** Prediction results of single-output models for LAI under different modal feature combinations.

Prediction pipeline	Model Feature	Stepwise		BP		SVR		RF		BNN	
		R2	RRMSE	R2	RRMSE	R2	RRMSE	R2	RRMSE	R2	RRMSE
LAI → LAI	NDRE	0.72	0.18	0.65	0.23	0.65	0.28	0.67	0.27	0.73	0.25
	CVI	0.55	0.32	0.41	0.32	0.59	0.34	0.41	0.42	0.46	0.35
	SV_COR	0.70	0.20	0.65	0.22	0.56	0.43	0.66	0.28	0.74	0.24
	F1	0.70	0.21	0.77	0.19	**0.73**	0.19	0.70	0.21	0.79	0.22
	F2	0.79	0.17	0.83	0.21	0.44	0.36	0.76	0.23	**0.83**	0.20
	F3	0.74	0.19	0.49	0.37	0.67	0.41	0.44	0.41	0.68	0.46
	F4	**0.84**	0.15	**0.83**	0.16	0.65	0.28	**0.81**	0.21	0.80	0.15
LAI → LNA	NDRE	0.54	0.57	0.51	0.60	0.41	0.60	0.47	0.57	0.47	0.57
	CVI	0.42	0.60	0.40	0.63	0.26	0.67	0.32	0.64	0.20	0.74
	SV_COR	0.56	0.45	0.56	0.48	0.57	0.51	0.55	0.52	0.63	0.47
	F1	0.63	0.47	0.59	0.46	**0.60**	0.49	0.50	0.55	0.58	0.50
	F2	0.55	0.53	0.65	0.42	0.25	0.68	0.53	0.53	0.64	0.46
	F3	0.65	0.46	0.68	0.45	0.26	0.62	0.22	0.63	0.67	0.49
	F4	**0.66**	0.45	**0.69**	0.39	0.40	0.61	**0.58**	0.51	**0.69**	0.43

F1, combination of NDRE and CVI; F2, combination of NDRE and SV_COR; F3, combination of CVI and SV_COR; F4, combination of NDRE, CVI, and SV_COR. Underlined values indicate the highest accuracy in each row, and bold values indicate the highest accuracy in each column.

**Table 4:** Prediction results of single-output models for LNA under different modal feature combinations.

Prediction pipeline	Model Feature	Stepwise		BP		SVR		RF		BNN	
		R2	RRMSE	R2	RRMSE	R2	RRMSE	R2	RRMSE	R2	RRMSE
LNA → LNA	NDRE	0.74	0.37	0.59	0.44	0.52	0.49	0.54	0.49	0.60	0.46
	CVI	0.52	0.50	0.36	0.60	0.37	0.57	0.33	0.67	0.51	0.50
	SV_COR	0.76	0.35	0.74	0.37	0.65	0.43	0.67	0.41	0.52	0.50
	F1	0.54	0.49	0.51	0.52	**0.66**	0.42	**0.69**	0.41	0.69	0.39
	F2	0.73	0.37	0.69	0.40	0.35	0.58	0.50	0.51	0.59	0.46
	F3	0.36	0.62	0.34	0.65	0.46	0.72	0.36	0.62	**0.73**	0.37
	F4	**0.78**	0.33	**0.74**	0.35	0.38	0.56	0.54	0.49	0.64	0.43
LNA → LAI	NDRE	0.59	0.35	0.68	0.30	0.61	0.36	0.63	0.35	0.67	0.33
	CVI	0.45	0.58	0.44	0.59	0.27	0.48	0.35	0.54	0.44	0.42
	SV_COR	0.49	0.55	0.48	0.57	0.51	0.40	0.69	0.33	0.65	0.34
	F1	0.64	0.39	0.62	0.40	**0.68**	0.32	**0.69**	0.32	0.63	0.35
	F2	0.66	0.39	0.64	0.39	0.36	0.45	0.59	0.37	**0.68**	0.32
	F3	0.62	0.40	0.60	0.41	0.21	0.47	0.31	0.46	0.19	0.50
	F4	**0.64**	0.38	**0.65**	0.38	0.40	0.44	0.62	0.35	0.66	0.33

The bold values indicate the best accuracy.

From the perspective of feature modalities, when a single modality was used to predict LAI and LNA, spectral features and image features generally performed better than structural features. When two modalities were combined, the model prediction accuracy was further improved. When all three modalities were jointly used as inputs, all models except SVR achieved the best prediction results, indicating that multimodal feature fusion can effectively enhance the representation ability of models for wheat growth parameters and improve prediction performance.

From the perspective of machine learning models, stepwise linear regression and BP neural networks showed relatively good overall predictive performance. However, when a single-output model trained for one target was used to predict the other target, the prediction accuracy was generally low. In other words, models trained with LAI as the target variable could not effectively predict LNA, and vice versa. This indicates that, although LAI and LNA exhibit a certain correlation in the observed data, single-output models still cannot fully exploit the intrinsic relationship between them, thereby limiting the transferability of the model across different target parameters.

**Figure 6:**
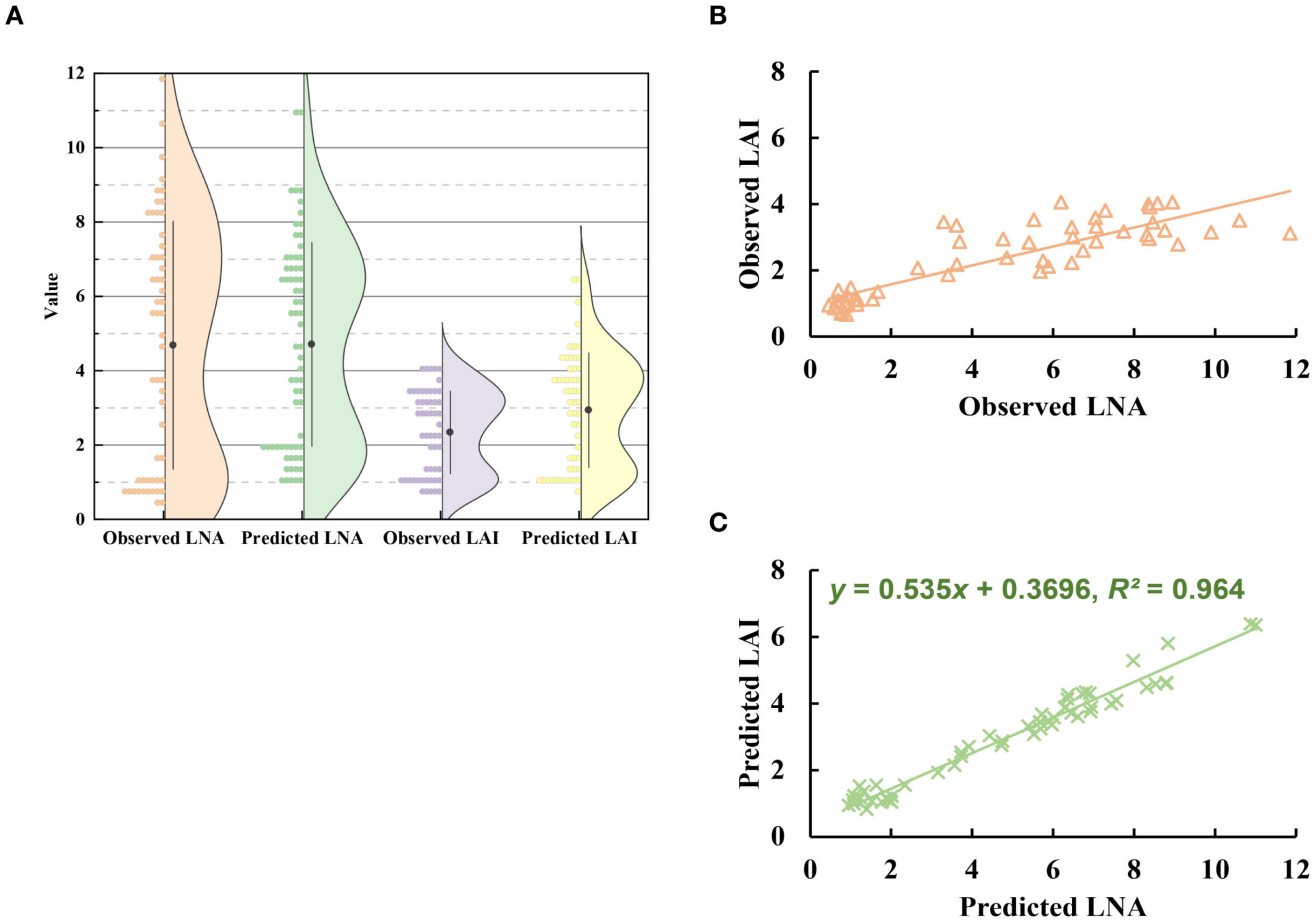
Comparison of observed and predicted distributions and correlations of LAI and LNA based on the optimal single-output model. **(A)** Distribution comparison between observed and predicted LAI and LNA. **(B)** Correlation between observed LNA and observed LAI. **(C)** Correlation between predicted LNA and predicted LAI.

To further analyze the limitations of single-output models, the model with the best predictive performance was selected, and its prediction results were compared with the actual observations. **Figure 6A** shows the distribution differences between the predicted and measured values. It can be seen that the predicted and measured distributions are relatively close for LNA, whereas a certain deviation still exists for LAI. **Figures 6B, C ** compare the correlations between the measured values and the correlations between the predicted values for LNA and LAI, respectively. The results show that the correlation between the predicted values is significantly higher than that in the observed data. This indicates that, although single-output models can fit each individual parameter separately, they cannot accurately preserve the association structure between LAI and LNA in real samples, and thus the prediction results fail to fully reflect the true variation relationships between different parameters.

### 3.4 Analysis of prediction results of dual-output models based on different modalities

**Table 5** presents the prediction results of different dual-output models for LAI and LNA. Overall, the Dual output BNN outperformed the Dual output BP, indicating that the introduction of Bayesian inference helps improve the model’s ability to learn multimodal features in joint prediction tasks. For LAI, the Dual output BNN achieved the best performance under full-modal feature fusion, with (*R^2^*) and RRMSE reaching 0.85 and 0.18, respectively. For LNA, the best performance was obtained when NDRE and CVI were fused, with (*R^2^*) and RRMSE of 0.71 and 0.39, respectively, while the result under full-modal fusion was very close. This result indicates that, although multimodal features can provide richer information, simply feeding all modal features directly into the model does not always lead to a continuous improvement in predictive performance.

**Table 5:** Prediction results of dual output models for LAI and LNA under different modal feature combinations.

Model Feature	Dual output BP				Dual output BNN				Improved dual output BNN			
	LAI		LNA		LAI		LNA		LAI		LNA	
	R2	RRMSE	R2	RRMSE	R2	RRMSE	R2	RRMSE	R2	RRMSE	R2	RRMSE
NDRE	**0.69**	0.26	0.55	0.47	0.67	0.24	0.53	0.52	0.77	0.23	0.61	0.45
H_mean	0.14	0.50	0.11	0.83	0.13	0.43	0.12	0.68	0.22	0.54	0.16	0.71
SV_COR	0.34	0.44	0.35	0.71	0.54	0.32	0.26	0.62	0.54	0.32	0.26	0.62
F1	0.47	0.34	0.15	0.65	0.55	0.32	0.26	0.62	0.55	0.32	0.26	0.62
F2	0.59	0.30	**0.63**	0.43	0.78	0.22	**0.71**	0.39	0.85	0.18	0.74	0.37
F3	0.22	0.51	0.14	0.75	0.32	0.39	0.35	0.58	0.49	0.33	0.58	0.47
F4	0.63	0.28	0.61	0.55	**0.85**	0.18	0.70	0.40	**0.89**	0.15	**0.77**	0.35

The bold values indicate the best accuracy.

In comparison, the Improved dual output BNN further outperformed the Dual output BNN. Under full-modal feature fusion, it achieved the best prediction accuracy for both LAI and LNA, with (*R^2^*) values increased to 0.89 and 0.77 and RRMSE values reduced to 0.15 and 0.35, respectively. This result indicates that, after introducing the ESM strategy into the dual-output Bayesian neural network, the model was able to more fully learn the complementarity and consistency among different modalities, thereby reducing the effects of information redundancy and information conflict on the prediction results and further improving the joint prediction performance.

**Figure 7:**
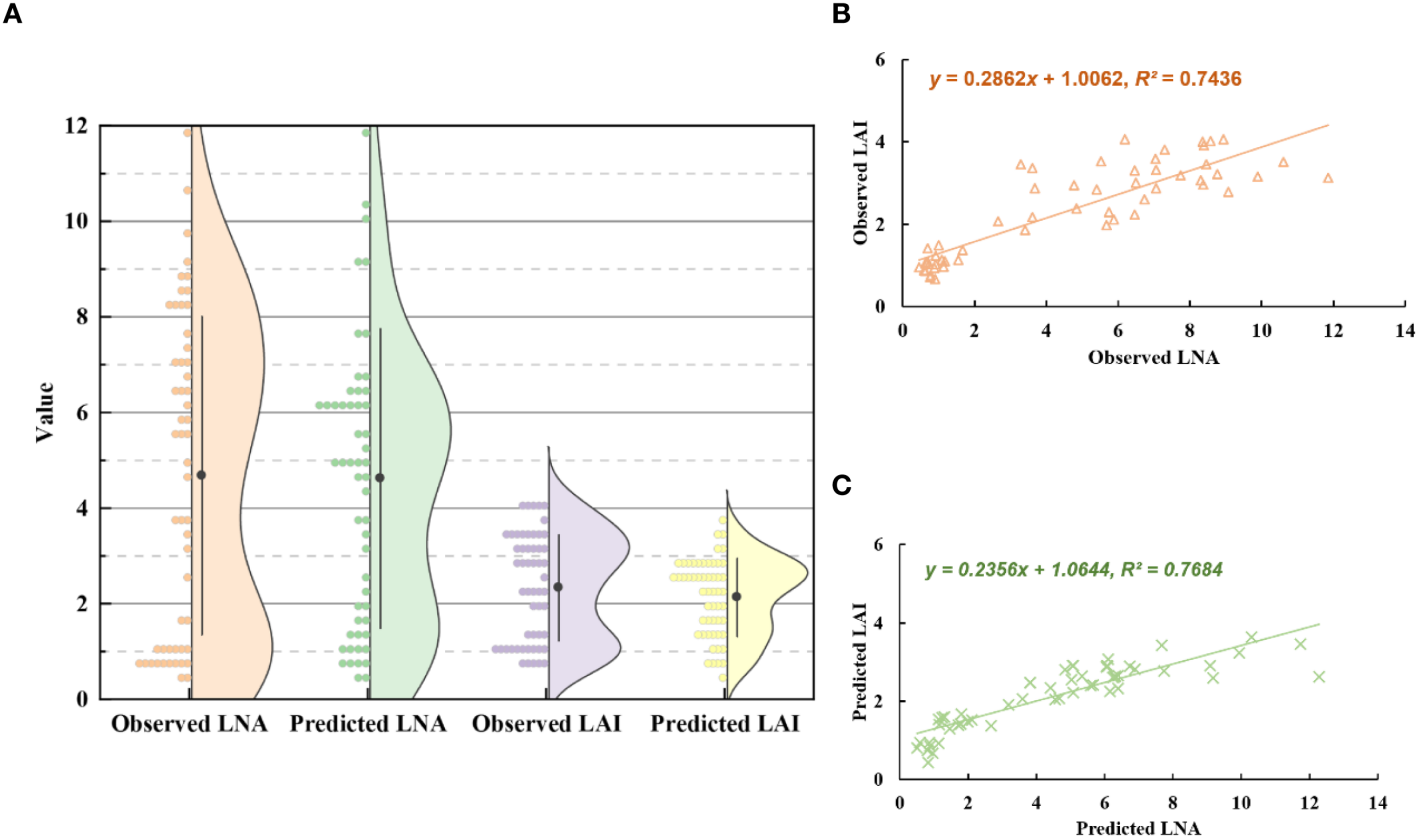
Comparison of observed and predicted distributions and correlations of LAI and LNA based on the optimal dual output model. **(A)** Distribution comparison between observed and predicted LAI and LNA. **(B)** Correlation between observed LNA and observed LAI. **(C)** Correlation between predicted LNA and predicted LAI.

**Figure 7** shows the simultaneous prediction results of LAI and LNA obtained by the Improved dual output BNN. Compared with the single-output model, the improved dual-output model not only achieved higher prediction accuracy but also produced prediction distributions that were closer to the actual observations. This indicates that, while improving predictive performance, the model was also better able to preserve the association structure between LAI and LNA in real samples, further verifying its effectiveness in multimodal dual-output prediction tasks.

### 3.5 Prediction uncertainty validation of the improved DO-BNN

To further verify the validity of the uncertainty estimated by the improved DO-BNN, repeated stochastic sampling predictions were performed for each sample in the test set. The mean of the repeated predictions was taken as the final predicted value, while the standard deviation of the predictions was used to characterize sample-wise predictive uncertainty. Furthermore, the absolute prediction errors of LAI and LNA were calculated, and the relationship between predictive uncertainty and absolute prediction error was analyzed.

**Figure 8:**
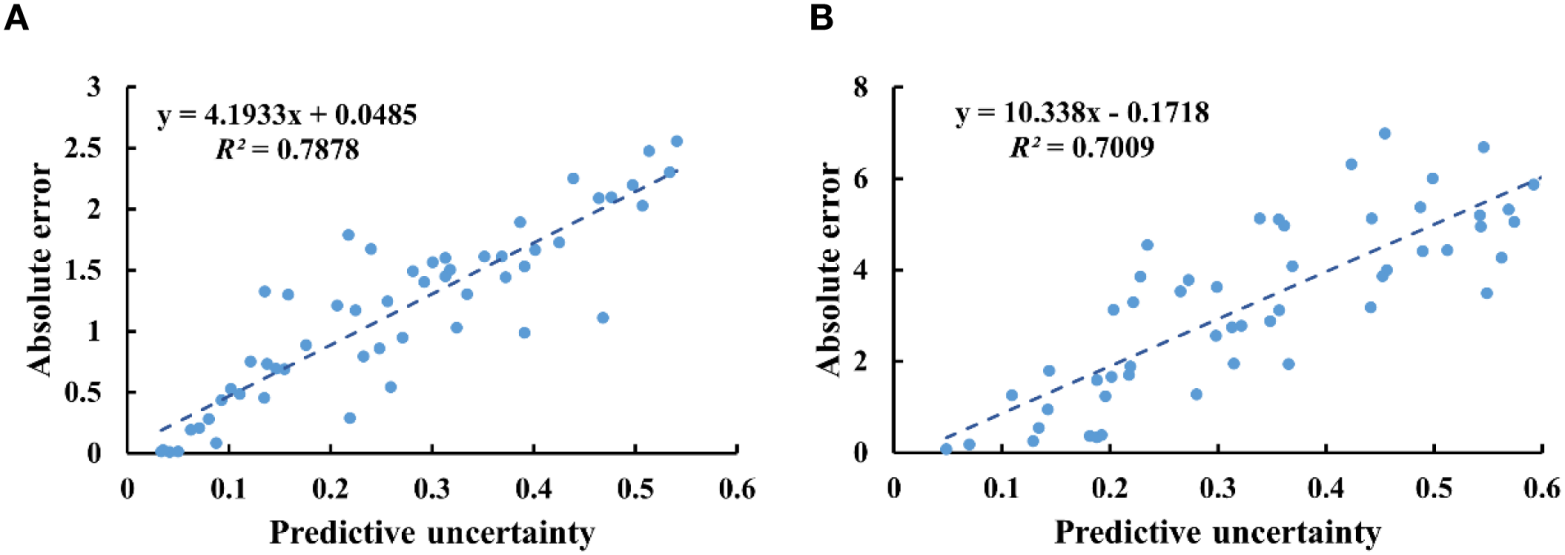
Relationships between predictive uncertainty and absolute prediction error. **(A)** LAI **(B)** LNA.

**Figure 8** shows the relationships between predictive uncertainty and absolute prediction error for LAI and LNA. It can be seen that, for both LAI and LNA, predictive uncertainty exhibited a clear positive correlation with absolute prediction error, indicating that samples with higher predictive uncertainty usually corresponded to larger prediction errors. This suggests that the uncertainty estimated by the improved DO-BNN can effectively reflect prediction risk at the sample level, rather than being merely an incidental output of the model. Combined with the results shown in **Figure 7**, it can be seen that the Improved dual output BNN not only improves the joint prediction accuracy of LAI and LNA and produces prediction distributions closer to the actual observations but also provides reliable information about prediction confidence. This further demonstrates that the proposed model has potential robustness and practical value for the collaborative prediction of wheat growth parameters under complex conditions.

### 3.6 Ablation study

To further quantify the contribution of the main components of the proposed framework, ablation experiments were conducted under the full-modal feature combination F4. Specifically, the single-output BNN with F4 was used as the baseline to evaluate the effect of independent modeling, the DO-BNN with F4 was used to assess the contribution of dual-output joint learning, and the improved DO-BNN with F4 was used to further evaluate the effectiveness of the Extreme Sample Mining (ESM) strategy. As shown in **Table 6**, the single-output BNN achieved R^2^/RRMSE values of 0.80/0.15 for LAI and 0.64/0.43 for LNA. After introducing the dual-output structure, the DO-BNN improved the R^2^ values to 0.85 for LAI and 0.70 for LNA, indicating that joint modeling helped the model exploit the intrinsic relationship between LAI and LNA. However, the RRMSE of LAI slightly increased from 0.15 to 0.18, suggesting that dual-output learning alone may introduce additional optimization difficulty when balancing the two prediction tasks. After further incorporating the ESM strategy, the improved DO-BNN achieved the best overall performance, with R^2^/RRMSE values of 0.89/0.15 for LAI and 0.77/0.35 for LNA. Compared with the DO-BNN without ESM, the improved DO-BNN increased the R^2^ values by 0.04 and 0.07 for LAI and LNA, respectively, while reducing the RRMSE values from 0.18 to 0.15 for LAI and from 0.40 to 0.35 for LNA. These results demonstrate that the dual-output structure can improve the collaborative representation of LAI and LNA, while the ESM strategy further enhances the model’s ability to learn from difficult and extreme samples, leading to more stable and accurate joint prediction.

**Table 6:** Results of ablation study.

Model setting	LAI		LNA	
	R2	RRMSE	R2	RRMSE
Single-output BNN + F4	0.80	0.15	0.64	0.43
DO-BNN + F4	0.85	0.18	0.70	0.40
Improved DO-BNN + F4	0.89	0.15	0.77	0.35

## 4 Discussion

### 4.1 Prediction of wheat LAI and LNA based on multimodal features

Previous studies have shown that extracting the absorption characteristics of vegetation in the red band and the reflection characteristics in the near-infrared band using multispectral remote sensing can effectively estimate wheat LAI and LNA (Zu et al., 2024; Sun et al., 2022). However, canopy spectral reflectance is influenced not only by leaf biochemical components, but also by canopy structure, leaf arrangement, and observation geometry, and therefore exhibits pronounced anisotropic characteristics (Kong et al., 2022; Liu et al., 2024). Under such conditions, prediction models built solely on spectral features cannot adequately characterize the complex physiological and structural states of crops, which is one of the main reasons for the limited stability of such models. In addition to spectral information, crops under different nutritional conditions also exhibit differences in stem and leaf color and morphological characteristics, which can, to some extent, reflect crop growth vigor and physiological status (Zhang et al., 2024a, b). Therefore, further integrating image features on the basis of spectral features can help improve the comprehensive characterization of crop status by the model.

Based on this, this study further introduced depth images in addition to spectral and two-dimensional image features and enhanced the characterization of wheat canopy structural status by extracting canopy height- and volume-related features. The results showed that the inclusion of depth information effectively improved the prediction accuracy of LAI and LNA, indicating that structural features play an important complementary role in the inversion of growth parameters. Nevertheless, multimodal features do not necessarily lead to continuous performance improvement through simple concatenation. The results of this study showed that, in some cases, directly inputting all modal features into the model did not yield the optimal results, suggesting that information redundancy, information conflict, and scale inconsistency may exist among different modalities, thereby increasing the difficulty of model learning and affecting prediction stability. Therefore, the key to multimodal prediction lies not only in introducing more data sources, but also in constructing a reasonable fusion strategy to fully exploit the independence and complementarity of information from different modalities. The Improved DO-BNN achieved better results under the full-modal condition, further demonstrating that targeted feature fusion and sample learning strategies can more effectively exploit the advantages of multimodal information in the collaborative prediction of wheat LAI and LNA.

### 4.2 Growth parameter prediction models based on machine learning

The selection and optimization of machine learning models are critical steps in crop growth parameter prediction (Cui et al., 2025). For parameters such as wheat LAI and LNA, which are jointly influenced by physiological status, canopy structure, and environmental conditions, different models differ markedly in feature representation ability, nonlinear fitting capability, and multi-target adaptability (Pourreza et al., 2025). Although traditional single-output models can, to some extent, achieve independent prediction of LAI or LNA, their modeling ability remains limited under scenarios involving multimodal inputs and collaborative prediction of multiple parameters (Gu et al., 2025).

In contrast, the Bayesian neural network adopted in this study showed stronger adaptability and robustness in multimodal and multi-parameter prediction tasks. Its core advantage lies in representing network weights as probability distributions and performing uncertainty modeling of the model parameters through Bayesian inference. In this way, the nonlinear representation ability of the neural network is retained while the robustness of the model to complex samples and limited-sample scenarios is improved. The results of this study showed that the Dual output BNN outperformed the Dual output BP overall, and the Improved dual output BNN with the ESM strategy further improved the joint prediction accuracy of LAI and LNA. This indicates that, during multimodal feature fusion, the BNN is more capable of adapting to information redundancy, conflicts, and scale differences among different modalities, while enhancing the representation of complex nonlinear relationships through probabilistic modeling. Future studies may further explore more efficient Bayesian approximate inference methods to reduce the computational cost of BNN training and inference. At the same time, structure-adaptive mechanisms or attention mechanisms could be incorporated to enhance the dynamic modeling ability of the model with respect to the importance of different modal features, thereby further improving the accuracy and efficiency of multimodal collaborative prediction.

### 4.3 Generalization ability of wheat multi-parameter prediction models

Different crop growth parameters usually exhibit significant physiological and statistical correlations; therefore, joint prediction of multiple parameters has become an important direction in crop growth monitoring research (Du et al., 2023). Existing studies on multi-parameter crop inversion have often focused on selecting sensitive bands or constructing specific vegetation indices, with the aim of achieving simultaneous monitoring of multiple growth parameters using single-source spectral information (Sun et al., 2022). For example, some studies combined Competitive Adaptive Reweighted Sampling (CARS) with Sobol sensitivity analysis to extract sensitive wavelengths for chlorophyll content and LAI, which were then used to build estimation models for rice canopy LAI and chlorophyll content (Jin et al., 2024). Other studies used multiple vegetation indices to invert several phenotypic parameters of sugar beet, including LAI, leaf chlorophyll and nitrogen contents, as well as canopy chlorophyll and nitrogen contents (Jay et al., 2017). These studies showed that the optimal vegetation index varies across parameters, and that inversion accuracy is also affected by factors such as observation angle. These findings indicate that a single vegetation index is generally insufficient to characterize multiple crop growth parameters stably and accurately at the same time, and that the generalization ability of such models is easily constrained by target parameter type and changes in observation conditions.

The results of this study similarly indicate that, although wheat LAI and LNA are correlated to some extent, their response relationships with different modal features are not entirely consistent. Even when a single-output model achieves good fitting performance for one parameter, it is still difficult to preserve the true association structure among different growth parameters simultaneously, which to some extent limits the model’s generalization ability in multi-parameter prediction tasks. In contrast, the ESM strategy proposed in this study, which is based on the idea of active learning, can more fully exploit the intrinsic relationship between LAI and LNA during joint modeling and enhance the model’s ability to learn from complex samples, thereby enabling more stable collaborative inversion of multiple wheat parameters. Nevertheless, the coupling mechanisms of different modal features in multi-parameter joint prediction still need to be further clarified. Future studies may further investigate the coupling relationships between multimodal features and multiple growth parameters in order to improve model interpretability and further enhance generalization ability under cross-year, cross-cultivar, and complex field conditions.

Although the proposed improved DO-BNN showed advantages in the collaborative prediction of wheat LAI and LNA, its generalization ability should be interpreted cautiously. First, the current dataset was collected from a single experimental site, and the model has not yet been validated under substantially different ecological regions, soil types, or climate conditions. Second, although multiple cultivars and management treatments were included, cultivar diversity remains limited compared with real production systems. Third, the total number of samples is still relatively small for deep learning models. These factors may constrain the generalization ability of the model. Therefore, the robustness and practical applicability of the proposed DO-BNN should be further evaluated using larger cross-region datasets covering more cultivars, soil backgrounds, climatic conditions, and field management practices.

## 5 Conclusion

To address the limitations in the collaborative prediction of LAI and LNA, including insufficient representation by single-modal information, the inability of single-output models to exploit the intrinsic relationships among parameters, and the limited stability of multimodal fusion, this study proposed an improved DO-BNN based on multimodal information to achieve the simultaneous prediction of wheat LAI and LNA. The main conclusions are as follows:

1. Spectral, two-dimensional image, and depth image features of wheat canopies under different cultivars, nitrogen treatments, and growth stages were extracted, and a height feature extraction method based on CHCM was proposed. The results showed that the canopy height information extracted after CHCM correction was more consistent with the measured values and could characterize wheat canopy structural features more accurately.
2. A DO-BNN based on multimodal features was constructed to achieve the simultaneous prediction of LAI and LNA. The results showed that multimodal feature fusion could effectively improve model prediction performance, and that the dual-output model was better able than the single-output model to preserve the true association structure between LAI and LNA.
3. An ESM strategy and a joint loss function were introduced into the DO-BNN. These components enhanced the model’s ability to learn modal complementarity and output relationships. The improved DO-BNN achieved R^2^ values of 0.89 and 0.77 for LAI and LNA, respectively.

## Data Availability

The raw data supporting the conclusions of this article will be made available by the authors, without undue reservation.
